# Prone Dynamic CT Myelography in Spontaneous Intracranial Hypotension: Diagnostic Need and Radiation Doses

**DOI:** 10.1007/s00062-023-01269-z

**Published:** 2023-03-03

**Authors:** Niklas Lützen, Enrique Barvulsky Aleman, Christian Fung, Juergen Beck, Horst Urbach

**Affiliations:** 1 Dept. of Neuroradiology, Faculty of Medicine, University Medical Center Freiburg, University of Freiburg, Breisacher Str. 64, 79106 Freiburg, Germany; 2 Dept. of Neurosurgery, Medical Center—University of Freiburg, Faculty of Medicine, University of Freiburg, Freiburg, Germany

**Keywords:** Orthostatic headache, Spinal CSF leak, Dural tear, Digital subtraction myelography, Spinal longitudinal extradural CSF collection

## Abstract

**Background and Purpose:**

The diagnostic work-up in patients with spontaneous intracranial hypotension (SIH) and spinal longitudinal extradural CSF collection (SLEC) on magnetic resonance imaging (MRI) comprises dynamic digital subtraction myelography (dDSM) in prone position for leak detection. Dynamic computed tomography (CT) myelography (dCT-M) in prone position follows if the leak is not unequivocally located. A drawback of dCT‑M is a high radiation dose. This study evaluates the diagnostic needs of dCT-M examinations and measures to reduce radiation doses.

**Methods:**

Frequency, leak sites, length and number of spiral acquisitions, DLP and effective doses of dCT‑M were retrospectively recorded in patients with ventral dural tears.

**Results:**

Of 42 patients with ventral dural tears, 8 underwent 11 dCT‑M when the leak was not unequivocally shown on digital subtraction myelography. The median number of spiral acquisitions was 4 (range 3–7) and the mean effective radiation dose 30.6 mSv (range 13.1–62.16 mSv) mSv. Five of eight leaks were located in the upper thoracic spine (range C7/Th1–Th2/3). Bolus tracking of intrathecal contrast agent in dCT‑M was used to limit the number and length of spiral acquisitions.

**Discussion:**

A dCT‑M in prone position to localize a ventral dural tear is needed in every fifth patient with a SLEC on MRI. It is typically needed when the leak is located in the upper thoracic spine and when patients have broad shoulders. Measures to reduce the radiation dose include bolus tracking or to repeat the DSM with adjusted positioning of patient.

## Introduction

Spontaneous intracranial hypotension (SIH) is an acquired disease resulting in orthostatic headache, which is in almost all cases caused by cerebrospinal fluid (CSF) leaks [1, 2]. Patients with distinct SIH signs on head magnetic resonance imaging (MRI, so-called head-positive) and predominantly ventral spinal longitudinal extradural CSF collection (so-called SLEC-positive) are suggestive to have a ventral dural tear which is typically located with prone dynamic digital subtraction myelography (dDSM) [3]. Ventral dural tears are often located in the upper thoracic spine and difficult to visualize with myelographic techniques, especially when patients have broad shoulders superimposing the spinal canal. In these situations, additional prone dynamic CT myelography (dCT-M) may be needed.

The dCT‑M as a technique to localize so-called high-flow CSF leaks was introduced in 2003 [4, 5]. At this time patients were lying flat on the CT table. With the use of a custom made tiltable wooden table patients can be placed in an upside down position which helps to control the intrathecal contrast flow toward a possible leak in the upper thoracic spine.

To date, only few data have been published on the radiation dose of dCT‑M studies in SIH patients. This article provides detailed data of how often dCT‑M, radiation doses and localization of the ventral leaks were acquired. As radiation exposure of dCT‑M is ~3 times higher compared to dynamic digital subtraction myelography [6], approaches to reduce radiation exposure are evaluated.

## Material and Methods

A retrospective study was carried out of patients fulfilling the diagnostic criteria of SIH who had been evaluated with dynamic subtraction myelography and dynamic CT myelography in prone position [1].

We restricted the time period to 2021–2022 in order to assure that dynamic digital subtraction myelography, which typically is the first invasive diagnostic measure was carried out with a state of the art flat detector unit (Siemens icono biplane, Siemens Healthineers, Erlangen, Germany).

Radiation exposures were recorded from the structured dose reports. The study was approved by the ethics committee (1249/22). Informed consent of the patients was obtained.

Dynamic CT myelography was performed on a 64-row multidetector CT scanner (Somatom Definition 64 AS; Siemens Healthineers, Erlangen, Germany). The patient was placed on a customized table in a prone and head down position. Spiral acquisition was typically started a few seconds after the beginning of the injection of 15 ml Iomeprol 300M (Imeron 300M, Bracco Imaging, Milano, Italy) with a speed of approximately 2 ml/s. The first spiral acquisition was in a caudo-cranial direction, subsequent acquisitions alternating in cranio-caudal and caudo-cranial directions. Collimation was 0.75 mm, rotation speed 0.3 s, 120 kV reference tube voltage, 280 mAs reference tube current, and four-dimensional automatic real-time dose adjustment technology served as default parameters (CareDose4D, Siemens Healthineers).

**Fig. 1:**
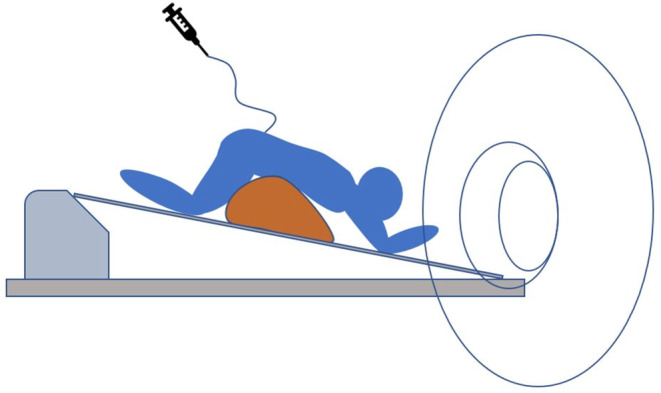
Schematic drawing of dynamic CT myelography in prone position

Dynamic CT myelography comprises the following steps:

1. Placing a spinal needle in the lumbar subarachnoid space either under fluoroscopy in lateral decubitus position or on the CT scanner table in prone position.
2. Connection of a 20-mL syringe filled with 15 ml of a nonionic contrast agent (300 mg/ml) to an extension tube and injection of 1–2 ml to confirm the intrathecal needle position.
3. Transport of the patient in lateral decubitus position from the flat detector unit to the CT scanner and placing the patient on a customized tiltable table in a prone and head down position with an additional pillow in order to compensate the lordosis of the lumbar spine (Fig. 1).
4. Acquisition of lateral CT scout of the spine covering the region of interest. The region of interest is determined using the MRI scans focusing on the SLEC sign and on osteophytes visible on MRI and the site suspicious of a ventral contrast egress in the epidural space on the dynamic myelograms.
5. Acquisition of 1–5 spiral CT scans during and after the continuous manual injection of contrast with the neuroradiologist wearing an X‑ray protective coat and standing behind a mobile acrylic lead glass shielding next to the gantry.

The approach is different from previous publications as the needle is placed under fluoroscopy and not in the CT scanner [7–9].

### Approaches for Radiation Dose Reduction

#### Bolus tracking of contrast agent in dCT‑M in prone position

**Fig. 2:**
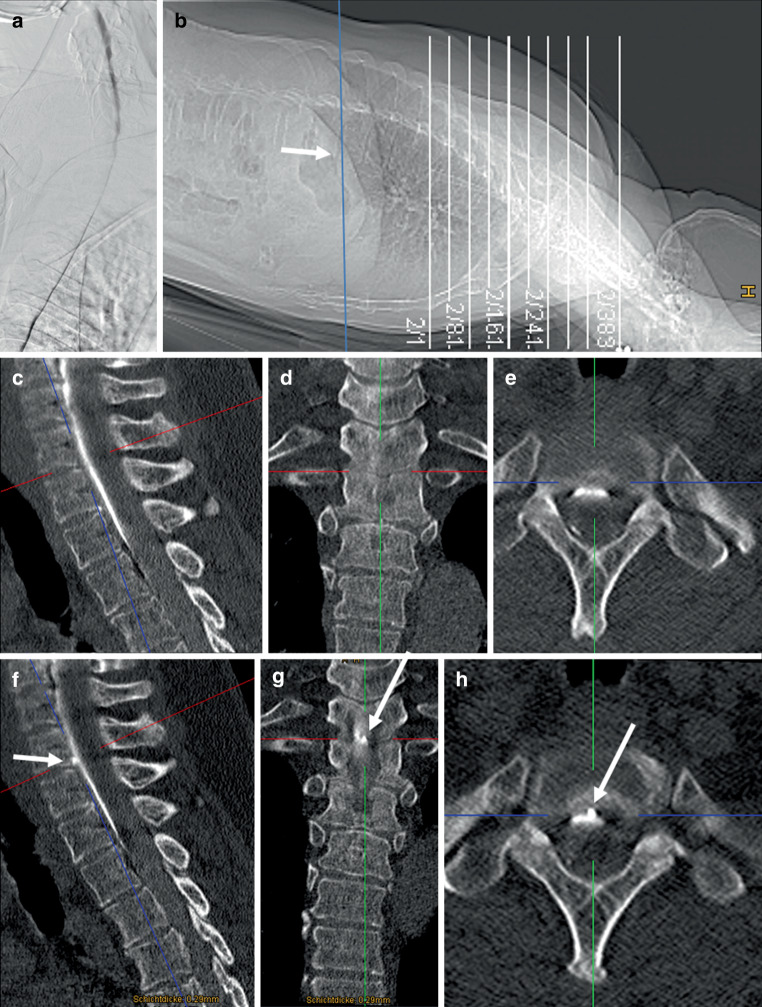
Dynamic digital subtraction myelography failed to detect a CSF leak (**a**). On a lateral scout, a monitor was placed in the mid-thoracic spine (**b**: *arrow*), scan range was from C6 to Th6. A second spiral acquisition in cranio-caudal direction showed no extrathecal contrast (**c**–**e**), while this was visible on a subsequent scan 20 s later at the level Th1/2 (**f**–**h**: *arrows*)

As it is unpredictable when the contrast column would overcome the thoracic kyphosis and reach the caudal border of the scanning range, we decided to include bolus tracking of contrast agent with the monitoring slice placed on the top of the thoracic kyphosis (Fig. 2b). The neuroradiologist in the CT room starts the injection of the contrast agent while the monitoring is started at the same time (one slice every second). Outside in the CT monitoring room, the scan is started by an assistant as soon as the contrast agent is visible intrathecally.

#### Shoulder Lift for Optimal, Overlay-free Imaging in DDSM

**Fig. 3:**
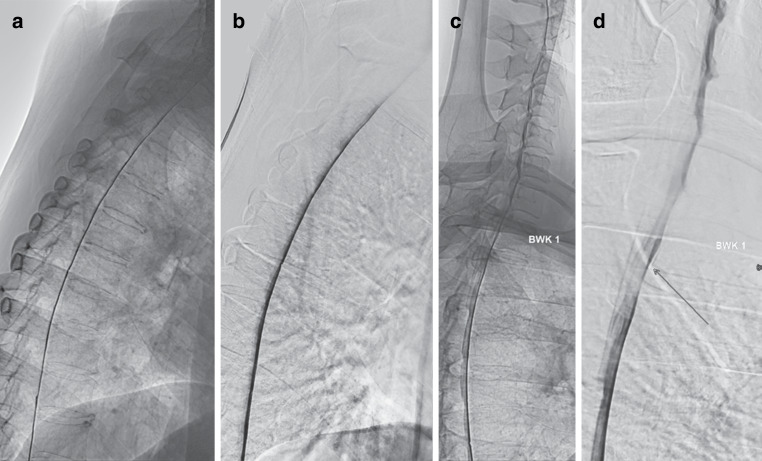
Prone dynamic digital subtraction myelography was repeated as the first examination poorly visualized the upper thoracic spine (**a**, **b**). Special attention was paid to elevate one shoulder on the second examination (**c**). It showed a tiny ventral leak at Th1/2 confirmed by surgery (**d**: *arrow*)

For optimal positioning of patients for dDSM in the angiography suite, they are placed in prone and “swimmer’s position”, with one arm extended toward the head and the other arm extended along the torso (Fig. 5). The shoulder with the arm extended forward is supported by a kidney cup and an inflatable pump wedge (Fig. 5) that raises the shoulder gradually until the spinal canal is no longer superimposed by the humeral head (Fig. 3c).

## Results

Out of 42 patients, who had ventral dural tears in an 18- month period 8 underwent 11 dCT‑M with a median number of 4 (range 3–7) spiral acquisitions and a mean effective radiation dose of 30.6 (range 13.1 to 79.8 mSv) (Table 1). In 34 patients, dDSM was able to show the exact site of leakage at the ventral aspect of the spine. In one patient, even dCT‑M did not succeed to find the exact site of leakage (patient #3).

**Table 1:** Patients with a ventral dural tear that was not detected by dynamic digital subtraction myelography (DSM) in prone position. In these patients, DSM was followed by dynamic CT myelography which revealed the leak, except in patient 3. The table shows the examination frequency, characteristics, localization of the leak, radiation doses and lists the use of bolus tracking of the contrast agent (in the right column)

Patient	Sex, age (years)	Leak location	kV mA/ref	Scanning range	Volume CT dose index (mGy)	Dose-length product (mGy×cm)	Effective dose (mSv); DLP × 0.017	Monitoring of contrast agent
1	m, 34	Th1/2	100 484/300	C4-Th5	21.5, 20.88, 21.59	339.2, 329.3, 340.4	17.59	+
	m, 35	Th 1/2	100 572/350	C7-Th6	25.41, 23.14, 25.23, 46.92	378.5, 344.5, 375.7, 529.8	28.22	+
2	m, 36	Th1/2	100 502/300	C4-Th6	22.3, 22.79, 22.39, 19.81	403.1, 410.0, 402.5, 565.8	31.55	+
3	m, 56	Not found	80 383/330	Th4‑9	8.02, 7.71, 8.59, 7.85, 6.97	136.5, 131.2, 53.8, 185, 160.5	13.00	+
	m, 56	Not found	100 369/300	Th5-10	16.39, 15.72, 16.39, 14.86, 12.57	208.6, 293.5, 306.2, 283.6, 308.7	26.28	+
4	m, 54	Th1/2	100 611/295	C6-Th6	12.43, 27.14, 25.45, 28.7	256.8, 517.9, 484.7, 486.8	48.73	+
5	f, 41	Th 9/10	100 292/495	Th5-L1	12.97, 12.39, 12.75	315,10 225,58 232,55	13,14	−
6	f, 64	Th12/L1	100 227/495	Th9-L1	10.08 9.91 10.08 9.46 9.37	180,37 177,22 180,37 214,08 211,96	16,39	−
7	m, 47	C7/Th1	120 511/288	C3-Th2	38.84 40.97 38.77	523,50 552,03 522,26	27,16	+
8	f, 59	Th 2/3	120 363/288	C5-Th8	16.87 27.59 27.59 33.67 27.06 27.67 26.98	283.3 562.2 561.9 350.6 620 607.2 618.8	62,16	+
	f, 59	Th 2/3	100 637/495	C4-Th7	14.75 28.3 27.5 28.2 28.16	290.6 558.6 541.6 557.7 555.2	43.5	+

Multiple numbers in the 6th and 7th column indicate the values of each CT scan

*f* female, *m* male, *C* cervical, *Th* thoracal, *L* lumbar, *mGy* milligray, *mSv* millisievert, *DLP* dose-length product

**Fig. 4:**
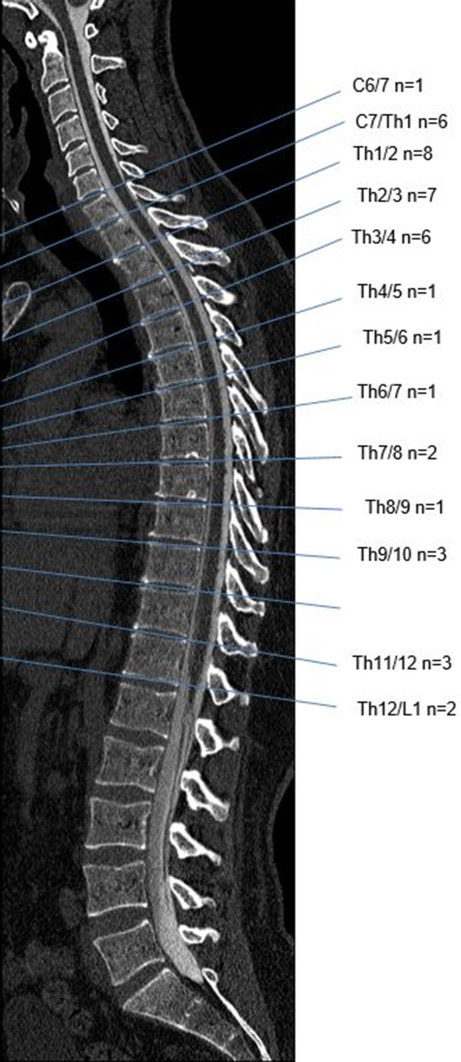
Location of 42 ventral leaks: 27 (64%) were in the upper thoracic spine. Prone dynamic CT myelography additional to dynamic subtraction myelography was performed in eight patients: five leaks were located at C7/Th1–Th2/3 (not highlighted separately in this Figure)

With respect to the entire cohort, 27 of 42 ventral leaks were located in the upper thoracic spine ranging from C7/Th1 to Th3/4. Of the additional dynamic CT‑M cohort, five of eight leaks were in this location (Table 1, Fig. 4).

Out of eight patients who underwent dCT‑M seven were performed with bolus tracking of contrast agent. Whether bolus tracking helped to limit the number and length of spiral acquisitions, cannot be proven.

**Fig. 5:**
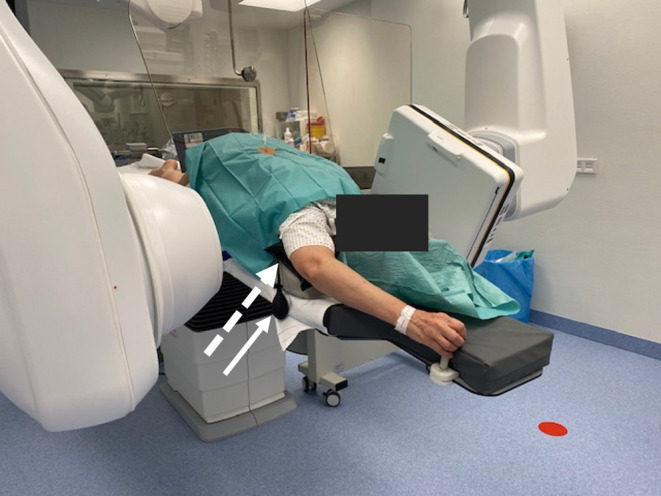
Patient in prone and swimmer’s position in the angiography suite, table tilted head deep. Right shoulder is lifted with a “pump wedge” (*dashed arrow*), gradually inflatable with a pump device (*arrow*)

Out of 34 patients who underwent dDSM 10 were performed with an additional “pump wedge” to lift one shoulder to increase image quality (Fig. 5).

## Discussion

In order to precisely locate ventral dural tears, additional dynamic CT myelography following dynamic digital subtraction myelography in prone position is needed in every fifth patient. The radiation dose is around threefold compared to digital subtraction myelography ([6–9]; Table 2) and measures to reduce it are desirable. The most effective way to avoid a high radiation dose is to avoid dynamic CT myelography and instead to repeat dynamic digital subtraction myelography. Therefore, the patient can be placed in swimmer’s position with one shoulder elevated using a so-called pump wedge so that it does not superimpose the spinal canal (Figs. 3, 4 and 5); however, this algorithm is only promising when the patients do not have broad shoulders or when spinal MRI suggests the leak to be located in the middle or lower thoracic spine. As two thirds of ventral dural tears are located in the upper thoracic spine, dynamic CT myelography in prone position will remain an indispensable tool in the work-up of SIH patients. Bolus tracking of contrast agent with placing the monitoring slice on the top of the thoracic kyphosis may help to reduce the number and length of spiral acquisitions although this cannot be proven from the small number of patients in this retrospective study.

**Table 2:** Radiation doses in dynamic CT myelography. For comparison, a dynamic digital subtraction myelography study is provided (bottom line)

**Study**	**Patients**	**Ventral leak location**	**Volume CT dose index (mGy)**	**Dose-length product (mGy × cm)**	**Effective dose (mSv)**
Luetmer 2003 [4]	*n* =4	n.r.	21.11	n.r.	n.r.
Luetmer 2012 [10]	*n* = 24	n.r.	n.r.	n.r.	n.r.
Thielen 2015 [7]	*n* = 14	3 cervical, 11 throracic	21.4 per scan with 3–6 scans	n.r.	70.6 (21.5–182.9)
Dobrocky 2018 [9]	*n* = 10	n.r.	107 (12–246)	1347 (550–3750)	24.3 (9.9–67.6)
Nicholson 2021 [6]	*n* = 33	n.r.	38.1 (10.2–103.7)	1184.9 (185.5–4848)	19.7 (3.2–82.4)
**Study**	**Patients**	**Ventral leak location**	**Fluoroscopy time (min)**	**Total kerma area product (mGy × cm**^**2**^)	**Effective dose (mSv)**
Nicholson 2021 [6]	*n* = 42	n.r.	4.7 (1.8–8.1)	34,053 (2932–88,374)	13 (2.6–31.7)

The values in the brackets indicate the ranges

*n* number, *n.r.* not reported, *mGy* milligray, *mSv* millisievert

Use of iterative reconstructions in CT examinations is a common means to reduce radiation dose [11]. Whether it is helpful to reduce the tube voltage to better use the iodine contrast or to use dual energy CT are further open questions [12].

## Limitations

We are aware that a retrospective study with changing the technique and scanning parameters over the time is far away from a controlled trial; however, the need to exactly locate ventral dural tears in patients with spontaneous intracranial hypotension is evident and many leaks have been missed for years [13, 14]. Thus, it is important to report on the technical challenges associated with the work-up of these patients.

## Conclusion

The use of dCT‑M has a high radiation dose but is sometimes indispensable in the search for ventral dural leaks in SIH patients. Its usage should be limited whenever possible by the less dose-intensive dDSM, where special positioning techniques of the patient may help to increase detectability of leaks. In addition, bolus tracking of contrast agent is an approach to potentially reduce the number of CT scans and therefore radiation dosages.
